# Implementation and Evaluation of Therapeutic Online Coaching Using Habit Reversal Training in Children With Tourette’s Disorder – A Pilot Study

**DOI:** 10.3389/fpsyg.2021.780539

**Published:** 2021-11-23

**Authors:** Paula Viefhaus, Julia Adam, Hildegard Goletz, Katrin Woitecki, Manfred Döpfner

**Affiliations:** ^1^School of Child and Adolescent Cognitive Behavior Therapy (AKiP), Faculty of Medicine and University Hospital Cologne, University of Cologne, Cologne, Germany; ^2^Department of Child and Adolescent Psychiatry, Psychosomatics and Psychotherapy, Faculty of Medicine and University Hospital Cologne, University of Cologne, Cologne, Germany

**Keywords:** videoconference, telehealth, blended therapy, habit reversal training (HRT), Tourette’s disorder

## Abstract

Cognitive-behavioral interventions can be difficult to implement in daily routine, which is often essential for generalizing treatment effects to natural settings. Furthermore, there is a lack of adequate care options concerning habit reversal training for children with Tourette’s disorder. The objective of this study is to evaluate therapeutic online coaching *via* videoconferencing in the natural environment of children with Tourette’s disorder in addition to face-to-face therapy (blended therapy). Online coaching took place twice a week for a maximum of 12 weeks. In a single-case study (*n* = 5; patients aged 8–11 years), the first results were obtained for exploratory purposes, especially with regard to the feasibility and reduction of symptoms and impairment. Various outcome measures were assessed (severity of symptoms, impairment, practical implementation, and satisfaction). Despite some principal limitations, the findings provide first hints that blended therapy is feasible and improves symptoms in some children with tics.

**Clinical Trial Registration:** [https://clinicaltrials.gov/], identifier [DRKS00017199].

## Introduction

Tics are defined as involuntarily sudden, rapid movements or vocal expressions that are often preceded by a premonitory urge (American Psychiatric Association, 2013). Cognitive-behavioral treatment (CBT), especially habit reversal training (HRT), is strongly recommended as an initial treatment for tic disorders by the European Clinical Guidelines (Verdellen et al., 2011), the Canadian Guidelines (Steeves et al., 2012), and the Clinical Guidelines of the American Academy of Neurology (Pringsheim et al., 2019). The aim of HRT (Azrin and Nunn, 1973) is to enable patients to gain control over their tics and to manage the respective urges. The most important components of this treatment are awareness training for tics and competing response training, according to which the patient learns to initiate a response to the urge which is incompatible with the tic (e.g., to strengthen the antagonist muscles), therefore controlling the tic.

A recent meta-analysis found a medium-sized effect of HRT on tic symptoms (McGuire et al., 2014), and according to the American Psychological Association’s Division 12 Task Force criteria, HRT is classified as a well-established treatment (Cook and Blacher, 2007). A large randomized controlled trial [Comprehensive Behavioral Intervention for Tics (CBIT), Piacentini et al., 2010] examining the effects of HRT plus functional interventions (eight sessions) as compared to education and supportive psychotherapy (eight sessions) found a significant treatment effect on tic symptoms in the HRT group, with a medium effect size.

The efficacy of a similar German treatment program for children and adolescents with chronic tic disorders (Therapieprogramm für Kinder und Jugendliche mit Tic-Störungen, THICS; Woitecki and Döpfner, 2015) was evaluated in two studies using within-subject designs (Woitecki and Döpfner, 2011; *n* = 16; Viefhaus et al., 2018; *n* = 27). The program was found to be effective in reducing tics and increasing the sense of controllability of tics.

Despite the established efficacy of HRT and the finding that it is welcomed by families due to its few to no side effects, the availability of such treatment is still limited (Cuenca et al., 2015). Moreover, it was found that families often have the perception that health professionals have only limited knowledge of tic disorders and their treatment (Cuenca et al., 2015). Furthermore, long travel distances to a specialized therapist often pose a huge burden for families (Himle et al., 2010). Therefore, many authors conclude that a wider training and greater availability of behavioral treatments are needed (e.g., Himle et al., 2010, 2012; Cuenca et al., 2015).

Due to the limited availability of HRT/CBIT-trained therapists, some adaptations of treatment delivery have been made to make it more easily accessible for a larger number of patients. One strategy for the dissemination of HRT is to provide it *via* telehealth approaches (cf. Himle et al., 2010). Telehealth uses telephone, internet and videoconference to provide psychological interventions, and was found to be effective in adolescents and young adults with different disorders, especially depression and anxiety (Hollis et al., 2017). Particularly in these challenging times of the COVID-19 pandemic, the provision of therapy using telehealth is of great importance.

One telehealth approach in tic patients consists of the therapist-guided online self-help format evaluated by Andren et al. (2019). In their study, patients (8–16 years, *n* = 23) were randomized either to HRT or to exposure with response prevention (ERP), both under online conditions. For both interventions, reduced tic-related impairment, parent-rated tic severity and improved quality of life were demonstrated, but only for ERP was a significant improvement in clinician-rated tic severity found (within-group effect sizes: ERP: 1.12; HRT: 0.50). An advantage of this approach is that it requires less therapist time per patient than regular face-to-face therapy.

Another promising approach is to deliver HRT *via* videoconference. This also has the advantage of eliminating travel requirements, and patients living in remote and underserved areas far away from specialized therapists are given the opportunity to receive an individualized CBT treatment. In a single-case study (*n* = 3; 11–17 years), Himle et al. (2010) delivered HRT *via* videoconference (eight sessions) and found a significant tic reduction (primary outcome: home-based video-recorded tic frequency rating). In addition, in a randomized controlled trial (8–17 years; *n* = 20; eight sessions), Himle et al. (2012) compared videoconference with face-to-face delivery and found tic reduction in both groups, with no between-group differences [primary outcome: Yale Global Tic Severity Scale (YGTSS)]. Ricketts et al. (2016) randomized patients (8–17 years; *n* = 20) to videoconferencing (received at home) or a waiting-list control group and found a greater tic reduction in the videoconference group (primary outcome: YGTSS). Furthermore, these studies found that participants and their parents were satisfied with the delivery modality of videoconferencing and reported a good therapeutic relationship (Himle et al., 2010, 2012; Ricketts et al., 2016).

Even though research has yielded promising findings regarding videoconferencing, more studies are needed, especially as previous studies only assessed either clinical ratings (Himle et al., 2010, 2012; Ricketts et al., 2016) or parent ratings (Ricketts et al., 2016). Given findings of only moderate correlations between parent reports and self-reports of tic symptoms (Döpfner and Görtz-Dorten, 2017), the assessment of tic symptoms based on self-reports may be highly important. Moreover, to verify whether learned HRT strategies are generalized to patients’ situation at home and used in their daily routine, it is desirable to conduct daily observations at home. As patients and families often state that besides reducing or stopping tics, a further desired outcome is to gain a sense of control over tics (Cuenca et al., 2015), it is also of great importance to assess controllability; however, this was not assessed in previous studies.

To date, no studies have examined the effects of the combination of face-to-face therapy and video-delivered therapy in children and adolescents with tics. Such a blended therapy approach has the advantage of reducing travel requirements while still offering direct contact with the therapist. Moreover, CBT interventions often struggle with problems of implementation into daily routine, which may be essential in order to generalize treatment effects from training during face-to-face therapy to everyday life, as the patients are required to practice HRT in each situation in which a tic occurs. Online coaching *via* videoconference offers a high flexibility and can be conducted in critical situations at home (e.g., homework situation) or at certain times when tics occur frequently (e.g., during an increase of tics after school or in the evening). Furthermore, due to the elimination of travel requirements, a high frequency of sessions (multiple times per week) is possible and the length of the sessions can be better adapted to the needs and the particular treatment phase of the patient (e.g., at the beginning, less time for practicing may be required than later on).

Therefore, the aim of this study is to implement and evaluate a therapeutic online coaching intervention using HRT in a naturalistic setting. Online coaching is conducted in addition to face-to-face therapy (blended therapy) and focuses on self-awareness training and the training of a specific competing response for each tic. The patient is instructed by the therapist, *via* videoconference, to practice and implement exercises at home especially in situations where tics increase.

In this proof-of-concept study, we applied a single-subject design in order to answer the following questions: (1) can home-based HRT *via* online coaching be successfully implemented (e.g., regarding the identification of motor tics and vocal tics as well as transmission quality? Is the transmission quality sufficient)? (2) Does home-based HRT *via* online coaching reduce tic symptoms and functional impairment and increase the sense of tic controllability? (3) Is online coaching experienced as helpful by patients, parents, and therapists?

## Materials and Methods

### Inclusion Criteria

Children and adolescents were eligible to take part in the study if they were aged 8–17 years and had been diagnosed with Tourette’s disorder (F95.2) according to the ICD-10 criteria (World Health Organization, 1992). Further inclusion criteria were (1) at least moderate severity of tic symptoms, as measured by a YGTSS Total Tic Score > 13 (cf. Piacentini et al., 2010); (2) tic disorders are the primary diagnosis; (3) tics occur in the domestic setting and can be observed audiovisually; (4) CBT and HRT in an outpatient setting are indicated; (5) patients and parents agree to HRT *via* online coaching (informed consent) and families have Wi-Fi at home (6); IQ of the patient >80. Exclusion criteria were (1) comorbid diagnosis of autism spectrum disorder; (2) parallel continuous behavior therapy at another outpatient unit or in private practice.

### Study Design and Treatment

The study was approved by the Ethics Committee of the University Hospital, Cologne and was registered at the German Clinical Trials Register (Identifier: DRKS00017199). Informed consent was obtained from patients and their parents prior to inclusion in the study.

In a single-case study using a two-phase AB design (Kazdin, 2011; Levin and Evmenova, 2014), the effectiveness of HRT using additional online coaching (blended therapy) was tested. Tic symptoms were measured at six assessment time points (t0–t5). Two intervention phases were conducted: intervention phase (A) started with t0 and ended after 3 weeks with t1, and included psychoeducation and the first step of HRT, comprising the description of the tic symptoms (Woitecki and Döpfner, 2015). No online coaching took place during this phase. This was followed by a 12-week intervention phase (B) with assessment time points every third week (t2, t3, and t4), ending with the final assessment point at t5. During this intervention phase (B), HRT elements were trained in online coaching sessions. Depending on individual needs, additional face-to-face sessions (up to one weekly session, blended therapy) were conducted (e.g., relaxation methods and functional interventions), but no tic awareness and competing response training elements were conducted during these face-to-face sessions. The face-to-face sessions were carried out by pedagogues or psychologists with a Master’s degree who were undergoing 3–5-year training to become child and adolescent psychotherapists or were already licensed. The online coaching was conducted by a specialized licensed child and adolescent psychotherapist.

The intervention was based on the therapy program for children and adolescents with chronic tic disorders (THICS; Woitecki and Döpfner, 2015), which follows the components of HRT outlined by Azrin and Nunn (1973). The intervention phase (B) contained the training of tic reaction detection, training of perception of the premonitory urge, and the competing response training according to which the patient learns to initiate a response to the urge which is incompatible with the tic (for a description of competing responses see section “Participants”). In all children, situational influences regarding frequency of tics were identified as well as situations in which it was easier or more difficult to detect tics (for a detailed description of the settings see section “Discussion”). At the beginning, situations and actions were chosen in which tics occurred and tic detection and/or practicing the competing response was easier because patients were able to focus and concentrate on the tics. Later on, more difficult situations were chosen for practicing HRT (e.g., while watching a movie). Online coaching took place 2–3 times per week in addition to face-to-face therapy. The duration of each online coaching session was between 15 and 50 min. If possible, only one tic was processed within a 3-week block. It was possible to train a tic over several blocks until the selected tic symptom had been significantly reduced according to clinical judgment. Following this, further tics were trained. The online coaching was carried out until no further therapy was indicated according to clinical assessment or until a maximum of 12 weeks of online coaching was reached.

The technological implementation of the online coaching was realized in accordance with the guidelines for webcam-based telemental health of the German National Association of Statutory Health Insurance Physicians (Kassenärztliche Bundesvereinigung, 2020a).

The software Patientus GmbH or Arztkonsultation GmbH were used, which are certified programs and provide a secure videoconference regarding protection of data privacy (Kassenärztliche Bundesvereinigung, 2020b). Therapists used a computer and a C920 Pro HD Logitech webcam. Patients used their own laptops, tablets, computers with webcams, or smartphones. As one family had problems with their own technical devices, they were provided with a smartphone (SAMSUNG Galaxy A5).

### Statistical Analysis

For the analyses, only those scale values were included that met the criterion of <10% missing values. To draw inferences about the reliability of the effects, the two different adjacent conditions, intervention (A) and intervention (B) were analyzed by visual inspection with a special focus on changes in tic symptoms, sense of uncontrollability and impairment across phases regarding symptom and impairment level, trend and latency of change (Kazdin, 2011). Additionally, means and standard deviations were computed for both intervention phases. For a more differentiated description of the course of change during intervention phase (B), this phase was divided into the two further sub-phases online coaching 1 (OC1) and online coaching 2 (OC2). For OC1, a mean score was calculated using the measurements at t2 and t3; for OC2, a mean score using t4 and t5 was calculated. Effects were aggregated across five patients and effect sizes were then computed by calculating the difference between pre- and post-treatment divided by the initial standard deviation.

Based on the inclusion criteria of YGTSS Total Tic Score >13 for patients with Tourette’s disorder, a tic score below these cut-offs at the final assessment was interpreted as a clinical success. Furthermore, a percentage reduction of the YGTSS Total Tic Score of 25% was considered as a positive response (Jeon et al., 2013).

### Outcome Measures

The primary outcome measure was the *YGTSS*, a clinician-rated, semi-structured interview which starts with a checklist of all tics present during the past week. Motor and vocal tics are rated separately on five domains: number, frequency, intensity, complexity, and interference (each score from 0 to 5). The domains are summed up to a Total Motor Score and a Total Vocal Score (range of each score: 0–25). These two scores are added up to a Total Tic Score (range 0–50). Additionally, an Overall Impairment Rating is assessed (“none” to “severe,” range: 0–50). The YGTSS has shown satisfactory validity and reliability in several studies (Leckman et al., 1989; Walkup et al., 1992; Storch et al., 2005).

The *parent-rated Symptom Checklist for Tic Disorders* (SCL-TIC-P) is an integral part of the Diagnostic System for Mental Disorders according to ICD-10 and DSM-IV for Children and Adolescents (Döpfner and Görtz-Dorten, 2017). In the first part of the SCL-TIC-P, the presence of different motor and vocal tic symptoms is assessed using 13 items. For each tic, the frequency of occurrence (“not at all” to “constantly – every few minutes,” range 0–4) and intensity of present tics (“very mild” to “severe, irritates others,” range 1–4) is assessed for the last week. A tic symptom score (range 0–16) was calculated by multiplying the frequency and intensity ratings for each item and then adding the products and dividing by all given tics (13). The internal consistency of the SCL-TIC-P has been found to be satisfactory (α = 0.70 to α = 0.79; Döpfner and Görtz-Dorten, 2017). Additionally, one item of the SCL-TIC-P assesses the sense of controllability of tic symptoms on a 5-point scale (“1 = very low” to “5 = very high”). For the purpose of the present study, these items were pooled into sense of “uncontrollability” (“0 = no sense of uncontrollability” to “4 = very high sense of uncontrollability”). Furthermore, five items assess the impairment in different situations (e.g., at home and school) (“0 = no at all” to “3 = very high”); to calculate a total score, these items were summed up and divided by the given items.

The *self-rated Symptom Checklist for Tic Disorders* (SCL-TIC-S) has the same structure as the SCL-TIC-P and is designed for children aged 11 years and above. For the purpose of this study, it was completed by all children with the help of their therapists if needed. The internal consistency of the SCL-TIC-S is not satisfactory (α = 0.61 to α = 0.64), which is unsurprising given the heterogeneous symptoms of tic disorders and the fact that each item assesses a single tic or area of tics (Döpfner and Görtz-Dorten, 2017).

The *clinician-rated Diagnostic Checklist for Tic disorders* (SCL-TIC-C) is also part of the Diagnostic System for Mental Disorders according to ICD-10 and DSM-IV for children and adolescents (Döpfner and Görtz-Dorten, 2017). In the first part, the clinician rated 18 items regarding frequency of occurrence and intensity of present tics for the last week (for range and score calculation see SCL-TIC-P). Furthermore, the impairment is rated (five items cf. SCL-TIC-P). In the second part, the clinician rated the tic symptoms occurring during the assessment sessions (t0, t1, t2, t3, t4, and t5; 18 items; for range and score calculation see SCL-TIC-P). The internal consistency of the SCL-TIC-C is not fully satisfactory (α = 0.68 to α = 0.69; Döpfner and Görtz-Dorten, 2017).

*Daily tic ratings* were conducted by parents and patients. One to four individually defined tics as well as a total tic score (one global item) were rated regarding the daily frequency of occurrence (“not at all” to “constantly – every few minutes,” range 0–4) and intensity of the tics (“very mild” to “severe, irritates others,” range 1–4). For each tic, an individual tic score was calculated by multiplying the frequency and intensity ratings for each day, and a total tic score was calculated on the basis of the “total tic” item. As there was a high individual variability of the frequency of assessments during the course of the study, mean scores were calculated for each 3-week period (t0–t1; t1.1–t2; t2.1–t3; t3.1–t4; t4.1–t5).

The *tic frequency during tic training* was rated by the therapist and the patient during every online coaching session. Additionally, patients were instructed to do their HRT exercise on their own and to rate the tic frequency during each training session. The absolute frequency of the occurrence of the trained tic and the exact duration of the observation period were noted. As there was a high variability regarding the duration of the training sessions, for every session, a tic frequency score was calculated assuming a duration of 30 min, to ensure comparability between the measurements and between patients. When HRT for a trained tic ended and another tic was trained, the newly trained tic was observed. Furthermore, total tic frequency and intensity during the training were rated (cf. daily tic ratings).

As a global measure of treatment response, the *Clinical Global Impressions-Improvement scale* (CGI-I) was used (Guy and Bonato, 1970; Guy, 1976) at the final assessment time point (t5). Categories ranged from “very much improved” (1) to “very much worse” (7). The CGI-I shows satisfactory validity (Berk et al., 2008).

The *Questionnaire for Functional Impairment and Quality of Life of Children and Adolescents with Tic Disorders* (Volk, 2018) comprises a patient version (TiQuaLF-S) and a parent version (TiQuaLF-P) and were rated at t0 and t5. Both versions consist of 46 Functional Impairment items, which are rated on a 4-point Likert scale (range “0 = not at” all to “3 = especially”) pertaining to difficulties due to tics. After rating the Functional Impairment items, the rater assesses the Subjective Burden due to tics. Satisfactory internal consistencies were found for the Functional Impairment total scales (α = 0.89 to α = 0.95; Volk, 2018).

*Feasibility rating of the online coaching*. The clinician-rated feasibility inventory was developed for the purpose of this study. For every online coaching session, feasibility was rated on a 4-point-scale (“0 = not true” to “3 = very true”) with respect to whether image and audio quality were sufficient (transmission quality, three items) and whether HRT assignment *via* videoconferencing could be sufficiently observed (one item).

*Telepresence Questionnaire*. The Telepresence Questionnaire is based on the Telepresence in Videoconference Scale (Berthiaume et al., 2018). The patient-rated instrument includes six items to measure response to the videoconferencing method in order to assess how natural participants found the videoconference to be, and whether participants had the same feelings as they would if the therapist was physically present. Each item is rated on a scale from 0% (completely disagree) to 100% (completely agree). To evaluate telepresence, a mean score across the six items was calculated for each patient. The questionnaire was rated at post.

*Satisfaction with the online coaching*. For the present study, we developed an inventory to assess satisfaction, which was based on the Client Satisfaction Questionnaire adapted to Internet-based interventions (CSQ-I; Boß et al., 2016) and the “Therapy Evaluation Questionnaire” [German: Fragebogen zur Beurteilung der Behandlung (FBB); Mattejat and Remschmidt, 1998]. A clinician-report form (13 items) and a patient-report form (15 items) were developed. Each item is rated on a 4-point scale ranging from “0 = not true” to “3 = very true.” The instrument was administered after every third intervention week during intervention phase (B). For the evaluation of online coaching satisfaction, items were averaged across all assessment points.

## Results

### Participants

Patients were referred to the outpatient clinic of the School of Child and Adolescent Cognitive Behavior Therapy at the University Hospital Cologne. The patients were recruited for the study during the beginning of their routine care psychotherapy if inclusion criteria were fulfilled. All parents and children gave consent to participate in the study after the procedure had been fully explained. Participants were included between April 2019 and June 2019.

A total of five patients were included and all patients finished treatment per protocol. Table 1 shows the demographic and treatment characteristics of the sample. The participants’ age ranged from 8 to 11 years (*M* = 9;72, *SD* = 1;28); two of them were boys, and all patients met the criteria for Tourette’s disorder. No patients were receiving medication for tics or comorbid disorders. The mean number of face-to-face sessions with the patient per week (t1–t5) was 0.58, and on average, two online coaching sessions per week were conducted. In the clinical feasibility rating of the online coaching, a mean score of 2.48 (*SD* = 0.39) was found, indicating a good feasibility (maximum score = 3). Hence, in clinical rating, the image and audio quality were sufficient and HRT assignment *via* videoconferencing was sufficiently well observable; only some rare image and sound interferences occurred. However, in patient 4, the quality was often only just acceptable.

**TABLE 1:** Sample and treatment characteristics.

Patient	1	2	3	4	5	Overall
**Sample characteristics**						
Age (years)	9;6	8;0	11;3	10;10	9;9	*M* = 9;72 *SD* = 1;28
Gender	Female	Female	Male	Male	Female	40% male
Age at onset	5	5	5	3	3	4;24
Tic symptoms at beginning	Throat clearing, shoulder-elbow tic; coughing; tongue clicking; tongue over lips, eye blinking; head shaking; arm moving; facial grimacing	Coughing; making a noise while shaking the head; facial grimacing, head and shoulder shaking	Throat clearing; complex head and shoulder tic; simple shoulder tic; squeaking	Throat clearing; snuffling; mouth opening; eye blinking; grunting	Head shaking; shoulder movement; arm movement; facial grimacing; chuckling; squeaking; jumping	
Treated tic symptoms	Throat clearing (t1–t3) shoulder-elbow tic (t3–t5)	Coughing (t1–t3) making a noise while shaking the head (t3–t5)	Throat clearing (t1–t3) complex head and shoulder tic (t3–t5)	Throat clearing (t1–t4) snuffling (t1–t4) mouth opening (t4–t5)	Head shaking (t1–t4) noise (t4–t5)	
Comorbid disorder	None	Subclinical OCD symptoms	None	None	Dyscalculia	
**Treatment characteristics**						
Number of face-to-face sessions per week	t0–t1: 1 patient, 1 parent; t1–t5: 4 patient; t5–FU: 4 patient, 2 parent	t0–t1: 1 patient, t1–t5: 5 patient, 2 parent; t5-FU: 2 patient	t0–t1: 2 patient, parent 1; t1–t5: 7 patient; t5–FU: 3 patient, 1 patient and parent	t0–t1: 2 patient, 1 parent; t1–t5: 8 patient, 2 parent; t5–FU: 7 patient, 2 parent	t0–t1: 3 patient, parent 1; t1–t5: 11 patient, 1 parent; t5–FU: 6 patient, 2 parent	
Mean number of face-to-face sessions with the patient per week (t1–t5)	0.33	0.42	0.58	0.66	0.92	0.58 (0.21)
Transmission quality *M* (*SD*)	2.45 (0.71)	2.61 (0.63)	2.86 (0.31)	1.75 (0.47)	2.71 (0.63)	2.48 (0.39)

*M, mean, SD, standard deviation.*

Regarding the competing responses to the urge, all patients trained at least one response for a vocal tic and one for a motor tic, which was incompatible with the specific tic. For vocal tics with the mouth (coughing, throat clearing, and noise), the mouth was closed as soon as the urge to tic was recognized and a deep and relaxed breathing through the nose was conducted (breathing out for a little longer than breathing in). For the vocal tic with the nose (snuffling), a relaxed breathing in and out through the mouth was trained. The competing responses for motor tics were to strengthen the antagonist muscles. For example, the competing response for the shoulder-elbow tic was to tense the shoulders in a downward position and to press the hands onto the thighs while pressing the elbows toward the hips. The competing response for head shaking was to straighten and tense the neck muscles gently while lowering and tensing the shoulders. A possible competing response for mouth opening was to purse the lips together and push the teeth together. For complex tics (e.g., making a noise while shaking the head), a combination of these competing responses was conducted.

### Single-Case Analyses

#### Tic Symptoms, Impairment, and Uncontrollability (Measured at the Main Assessment Time Points)

**FIGURE 1:**
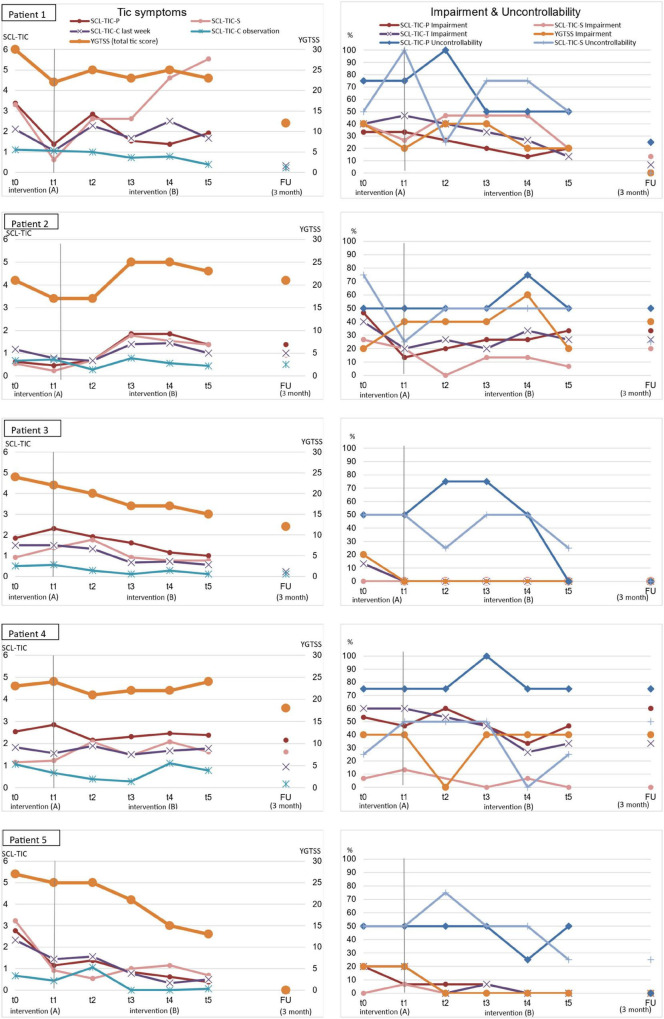
Tic symptoms, impairment, and uncontrollability during intervention (A) and intervention (B) for each patient.

Figure 1 shows the course of tic symptoms, impairment, and sense of uncontrollability during the interventions (A) and (B) for each patient. Regarding tic symptoms, a typical fluctuation is visible. During intervention phase (A), a reduction of tic symptoms was found in most patients. In some patients, and for some outcomes, the improvement during intervention phase (A) seems to be larger than the improvement in intervention phase (B) (e.g., patients 1 and 2, YGTSS Total Tic Score).

Regarding the primary outcome YGTSS Total Tic Score, a reduction was found during the intervention phase (B), especially in patients 3 and 5. While patients 1 and 4 showed no noteworthy change for YGTSS Total Tic Score during the intervention phase (B), a reduction was found during follow-up. Patient 2 showed an increase of the YGTSS Total Tic Score during intervention B, which can mainly be explained by two new severe and frequent tics.

In order to deal with the high fluctuation of the symptoms between the assessment points, mean scores of the phases (two assessments) were calculated for each patient for intervention phase (A) [*M*_Int.(A)_; t0 and t1], intervention phase OC1 (*M*_OC1_; t2 and t3), and intervention phase OC2 (*M*_OC2;_ t4 and t5), which are shown in Table 2. Furthermore, effect sizes from pre- to post-assessment and from pre-assessment to follow-up across all patients were calculated.

**TABLE 2:** Outcomes.

Patient	1				2				3				4				5				Overall					
	*M*_Int.(A)_ (*SD*)	*M*_OC1_ (*SD*)	*M*_OC2_ (*SD*)	FU	*M*_Int.(A)_ (*SD*)	*M*_OC1_ (*SD*)	*M*_OC2_ (*SD*)	FU	*M*_Int.(A)_ (*SD*)	*M*_OC1_ (*SD*)	*M*_OC2_ (*SD*)	FU	*M*_Int.(A)_ (*SD*)	*M*_OC1_ (*SD*)	*M*_OC2_ (*SD*)	FU	*M*_Int.(A)_ (*SD*)	*M*_OC1_ (*SD*)	*M*_OC2_ (*SD*)	FU	*M*_Int.(A)_ (*SD*)	*M*_OC1_ (*SD*)	*M*_OC2_ (*SD*)	FU	ES (t1–t5)	ES (t1–FU)
**YGTSS**																										
Total Tic Score	26 (4)	24 (1)	24 (1)	12 (0)	21 (0)	21 (4)	24 (1)	21 (0)	23 (1)	18.50 (1.50)	16 (1)	12 (0)	23.50 (0.50)	21.50 (0.50)	23 (1)	18 (0)	26 (1)	23 (2)	14 (1)	0 (0)	23.9 (1.91)	21.6 (1.88)	20.2 (4.31)	12.6 (7.2)	1.95	6.21
Impairment	15 (5)	20 (0)	10 (0)	0 (0)	15 (5)	20 (0)	20 (10)	20 (0)	5 (5)	0 (0)	0 (0)	0 (0)	20 (0)	10 (10)	20 (0)	20 (0)	10 (0)	5 (5)	0 (0)	0 (0)	13 (5)	11 (8)	10 (9)	8 (10)	0.48	0.48
**SCL-TIC-S**																										
Tic symptoms	1.96 (1.35)	2.62 (0)	5.08 (0.46)	0.23 (0)	0.38 (0.15)	1.23 (0.54)	1.46 (0.08)	0.92 (0)	1.15 (0.23)	1.35 (0.42)	0.77 (0)	0.23 (0)	1.19 (0.04)	1.77 (0.31)	1.85 (0.23)	1.62 (0)	2.08 (1.15)	0.77 (0.23)	0.92 (0.23)	0.00 (0)	1.35 (0.62)	1.55 (0.62)	2.02 (1.58)	0.60 0.59	−2.41	0.59
Impairment	1.00 (0.20)	1.40 (0)	1.00 (0.40)	0.40 (0)	0.70 (0.10)	0.20 (0.20)	0.30 (0.10)	0.60 (0)	0.00 (0)	0.00 (0)	0.10 (0.10)	0.00 (0)	0.30 (0.10)	0.00 (0)	0.10 (0.10)	0.00 (0)	0.10 (0.10)	0.00 (0)	0.00 (0)	0.00 (0)	0.42 (0.38)	0.32 (0.55)	0.28 (0.15)	0.20 (0.25)	0.76	0.63
Uncontrollability	3.00 (1)	2.00 (1)	2.50 (0.50)	0.00 (0)	2.00 (1)	2.00 (0)	2.00 (0)	1.00 (0)	2.00 (0)	1.50 (0.50)	2.00 (0)	1.00 (0)	1.50 (0.50)	2.00 (0)	0.50 (0.50)	2.00 (0)	2.00 (0)	2.50 (0.50)	1.50 (0.50)	1.00 (0)	2.10 (0.49)	2.00 (0.32)	1.70 (0.68)	1.00 (0.63)	0.55	1.10
**SCL-TIC-P**																										
Tic symptoms	2.38 (1)	2.19 (0.65)	1.65 (0.27)	0.23 (0)	0.54 (0.08)	1.27 (0.58)	1.62 (0.23)	1.38 (0)	2.08 (0.23)	1.77 (0.15)	1.08 (0.08)	0.23 (0)	2.69 (0.15)	2.23 (0.08)	2.42 (0.04)	2.15 (0)	1.96 (0.81)	1.12 (0.27)	0.50 (0.12)	0.00 (0)	1.93 (0.74)	1.72 (0.46)	1.45 (0.64)	0.80 (0.83)	0.23	0.88
Impairment	1.00 (0)	0.70 (0.10)	0.50 (0.10)	0.00 (0)	0.90 (0.50)	0.70 (0.10)	0.90 (0.10)	1.00 (0)	0.20 (0.20)	0.00 (0)	0.00 (0)	0.00 (0)	1.50 (0.10)	1.60 (0.20)	1.20 (0.20)	1.80 (0)	0.40 (0.20)	0.20 (0)	0.00 (0)	0.00 (0)	0.80 (0.46)	0.64 (0.55)	0.52 (0.48)	0.58 (0.73)	0.00	0.07
Uncontrollability	3.00 (0)	3.00 (1)	2.00 (0)	1.00 (0)	2.00 (0)	2.00 (0)	2.50 (0.50)	2.00 (0)	2.00 (0)	3.00 (0)	1.00 (1)	0.00 (0)	3.00 (0)	3.5 (0.50)	3.00 (0)	3.00 (0)	2.00 (0)	2.00 (0)	1.50 (0.50)	0.00 (0)	2.40 (0.49)	2.70 (0.6)	2.00 (0.71)	1.20 (1.17)	1.10	2.19
**SCL-TIC-C Interview**																										
Tic symptoms	1.58 (0.53)	1.97 (031)	2.08 (0.42)	0.33 (0)	0.97 (0.19)	1.03 (0.36)	1.22 (0.22)	1.00 (0)	1.50 (0)	1.00 (0.33)	0.64 (0.08)	0.22 (0)	1.69 (0.14)	1.69 (0.19)	1.72 (0.06)	0.94 (0)	1.89 (0.44)	1.17 (0.39)	0.42 (0.08)	0.00 (0)	1.53 (0.31)	1.37 (0.39)	1.22 (0.63)	0.50 (0.4)	0.50	2.28
Observed tics	1.08 (0.03)	0.86 (0.14)	0.58 (0.19)	0.22 (0)	0.69 (0.03)	0.53 (0.25)	0.50 (0.06)	0.50 (0)	0.53 (0.03)	0.19 (0.08)	0.19 (0.08)	0.11 (0)	0.86 (0.19)	0.33 (0.06)	0.94 (0.17)	0.17 (0)	0.56 (0.11)	0.53 (0.53)	0.03 (0.03)	0.00 (0)	0.74 (0.21)	0.49 (0.22)	0.45 (0.32)	0.20 (0.17)	1.44	2.12
Impairment	1.30 (0.10)	1.10 (0.10)	0.60 (0.20)	0.20 (0)	0.90 (0.30)	0.70 (0.10)	0.90 (0.10)	0.80 (0)	0.20 (0.20)	0.00 (0)	0.00 (0)	0.00 (0)	1.80 (0)	1.50 (0.10)	0.90 (0.10)	1.00 (0)	0.60 (0)	0.10 (0.10)	0.00 (0)	0.00 (0)	0.96 (0.55)	0.68 (0.57)	0.48 (0.41)	0.40 (0.42)	0.61	0.67

*Means of the intervention phase (A), intervention phase OC1, and intervention phase OC2 for each patient and across patients as well as effect sizes. M_Int.(A)_ = mean of score t0 and t1; M_OC1_ = mean of score t2 and t3; M_OC2_ mean of score t4 and t5; ES = (M_t1_–M_t5_)/SD_t1_ or = (M_t1–_M_FU_)/SD_t1_.*

Comparing the means of the phases [*M*_Int.(A)_; *M*_OC1_, *M*_OC2_], especially patients 3 and 5 showed improved or stable scores in all outcomes. Furthermore, patients 1 and 4 showed improved or stable scores except in self-rated tic severity (SCL-TIC-P) and clinician-rated SCL-TIC-C-Interview (patient 1), where an increase was found. In patient 2, a tic symptom increase was found during intervention phase OC2. Only regarding self-rated impairment can an improvement be seen in patient 2; the other ratings are stable (impairment and uncontrollability). With regard to the follow-up assessment, the symptoms (tics, impairment, and uncontrollability) were stable or even improved in all patients and outcome measures.

In terms of a descriptive comparison of the means across all patients (overall means), an improvement during the course of the intervention (A, OC1, and OC2) was found for all outcome measures. Comparing t1 and t5, effect sizes ranging from 0 to 1.95 were found, indicating no change to large improvement, with the exception of the self-rated tic score (SCL-TIC-S), where an effect size of −2.41 was found, indicating a large increase of tics. When comparing t1 and follow-up, effect sizes range from 0.07 to 6.21, indicating no change to very large effects.

Regarding subjective burden and functional impairment (rated at t0 and t5), descriptive improvements were found in patient ratings (functional impairment: *M*_t0_ = 0.28, *SD* = 0.16; *M*_t5_ = 0.17, *SD* = 0.24; subjective burden: *M*_t0_ = 0.52, *SD* = 0.36; *M*_t5_ = 0.20, *SD* = 0.28) and parent rating (functional impairment: *M*_t0_ = 0.31, *SD* = 0.17; *M*_t5_ = 0.17, *SD* = 0.12; subjective burden: *M*_t0_ = 0.68, *SD* = 0.48, *M*_t5_ = 0.55, *SD* = 0.44).

#### Daily Observations

**FIGURE 2:**
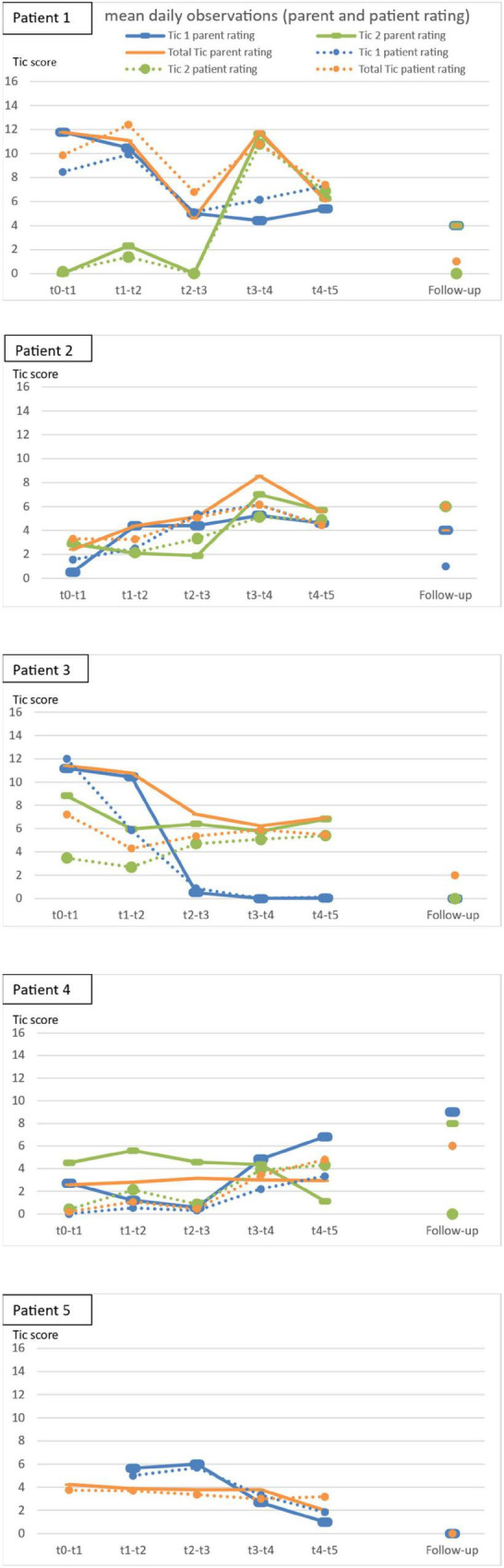
Mean tic scores of the daily observations. Follow-up consisted of only one observation. At the beginning, patient 5 observed different tics, which were no longer present; she therefore started observing the headshaking at t1, which was treated later on.

Figure 2 shows the course of the mean scores of the trained tics and the total tic score for the daily observations.

Patients 3 and 5 showed a reduction during the course of the intervention in most outcomes. The motivation of patient 2 was lacking, probably due to the occurrence of two new severe tics, the frequency of the online coaching, the frequent assessments and the confrontational nature of HRT. To avoid a further decrease in motivation, patient and therapist agreed to reduce the daily observations and ratings during tic training. In patients 2 and 4, an increase of observed tics can be found especially in self-rating, while in patient 1, no clear tendency is observable due to the high fluctuation.

#### Tic Frequency Rating During Tic Training

**FIGURE 3:**
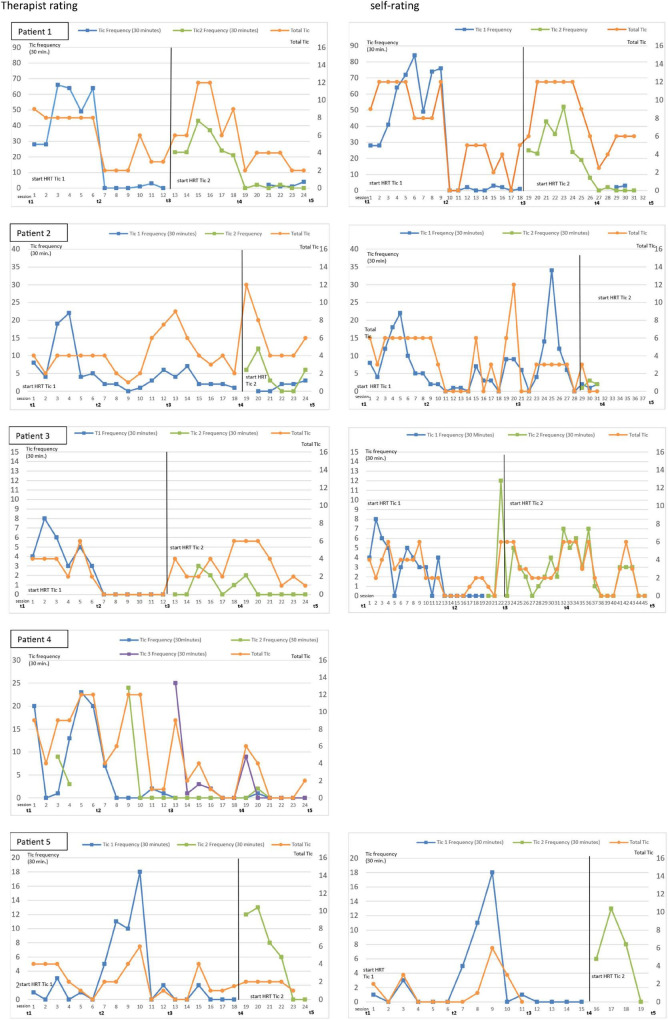
Tic frequency (30 min) in therapist and self-rating during tic training. Self-rating of patient 4 is missing.

Figure 3 shows the course of the tic frequency rated by the therapist and the patient during the training sessions. As some ratings by the patients are missing (not imputed) and the self-guided training (without therapist) was also assessed, therapist and patient ratings differ regarding the number of assessment points.

In patient 1, a large decrease in the trained tics (tic 1 and tic 2) can be observed (therapist and patient rating) after the start of the tic-specific habit reversal treatment. In patient 3, a large decrease is shown in tic 1. As tic 2 was only observed rarely and in specific situations (e.g., playing videogames), these situations were induced in order to increase tic frequency. Thus, tic 2 first increased and subsequently decreased after patient 3 had learned the competing response. As patient 3 had already started the observation before tic 2 was trained in the online coaching, the patient ratings began before the therapist ratings. As patient 5 initially had no or only very few tics in the training situation, the situation was modified to a situation where tics occurred more frequently (e.g., watching TV). Therefore, tic 1 first increased and then decreased after introducing the competing response.

Patient 4 developed ERP as a good strategy for himself (tic suppression instead of using a specific competing response); therefore, multiple tics were observed at once. At the end of the online coaching, he was able to suppress almost all tics for at least 30 min (and for even longer in face-to-face situations). In patient 2, no improvement was found.

Concerning the latency of change in four patients, an improvement regarding the specific tic was observed directly after introducing the competing response.

#### Clinical Significance

Table 3 shows the percentage of symptom reduction regarding the YGTSS Total Tic Score during treatment and follow-up phase and the percentage of patients who still fulfilled the inclusion criterion at t5 and FU (YGTSS Total Tic Score >13).

**TABLE 3:** Clinical significance: percentage of symptom reduction on YGTSS and percentage of patients with inclusion criterion fulfilled.

Patient	1	2	3	4	5	Overall
t1–t5 reduction	−5%	−10%	32%	0%	48%	13%
t1–FU reduction	45%	0%	45%	25%	100%	43%
Inclusion criterion still met at t5?	Yes	Yes	Yes	Yes	No	80% yes
Inclusion criterion still met at FU?	No	Yes	No	Yes	No	40% yes
CGI-I	1	3	2	2	2	2

*Inclusion criterion: YGTSS Total Tic Score > 13; CGI-I, Clinical Global Impressions-Improvement scale.*

A reduction of 25% in the YGTSS Total Tic Score is considered as a positive response (Jeon et al., 2013). During treatment, only two patients had a reduction of more than 25%, while three patients had a small increase or no change (−10 to 0%). During treatment and follow-up phase, four patients had a reduction of at least 25% while one patient showed no change (0%).

The inclusion criterion of YGTSS Total Tic Score >13 was still met in four patients at t5, while at follow-up, only two patients still met the inclusion criterion. Regarding CGI-I rating, one patient was rated as very much improved, three patients as much improved and for one patient no change was rated.

#### Treatment Satisfaction and Telepresence

Treatment satisfaction with the online coaching and telepresence are shown in Table 4. Patients 1, 3, and 5 as well as their therapists rated treatment satisfaction (range 0 = “not satisfied at all” to 3 = “very satisfied”) as very high during the course of the treatment (most ratings <2.5). In contrast, therapist and patient 2 rated treatment satisfaction lower (therapist: 1.15–2.38; patient: 1.40–1.67). While the therapist of patient 4 was mainly satisfied (1.31–2.50), the patient himself rated lower satisfaction (0.67–1.67).

**TABLE 4:** Treatment satisfaction and telepresence.

Patient	1				2				3				4				5				Overall *M* (*SD*)			
	t2	t3	t4	t5	t2	t3	t4	t5	t2	t3	t4	t5	t2	t3	t4	t5	t2	t3	t4	t5	t2	t3	t4	t5
Treatment satisfaction (therapist)	3.00	2.77	2.85	2.85	2.38	1.38	1.15	1.54	2.69	2.85	2.54	2.85	2.50	1.31	2.23	2.15	1.77	2.77	2.77	2.85	2.47 (0.46)	2.22 (0.80)	2.31 (0.69)	2.45 (0.59)
Treatment satisfaction (patient)	2.20	2.47	2.27	2.80	1.40	1.67	–	1.40	2.60	2.53	2.77	3.00	1.20	0.67	1.47	1.67	2.73	2.33	2.80	2.87	2.03 (0.70)	1.93 (0.79)	2.33 (0.62)	2.35 (0.75)
Telepresence (patient)				65.00				40.00				90.00				53.33				100.00				69.97 (25.01)

*M, mean. SD, standard deviation; patient 2 t4 is missing; range treatment satisfaction: 0 = not satisfied at all to 3 = very satisfied; interpretation telepresence: 0% = completely disagree to 100% = completely agree.*

Moreover, telepresence was rated by four patients, with at least 50% agreement (0% = completely disagree to 100% = completely agree). Telepresence was rated lower in patients 2 and 4 than in the other patients.

## Discussion

The aim of this single-case study was to develop and evaluate therapeutic online coaching applying the main components of HRT (self-awareness training and training of a specific competing response) in children and adolescents in a naturalistic setting (at home). Online coaching was conducted in addition to face-to-face therapy (blended therapy), and patients were instructed *via* videoconference to practice HRT exercises at home, especially in situations where tics increase. Multiple outcome measures were assessed, including ratings of clinicians, parents, and patients as well as behavioral observations.

Regarding tic symptoms, a typical fluctuation over the course of the treatment and the subsequent follow-up was found. The reduction of tic symptoms during the intervention phase (A) in most patients is noteworthy and might be partly explained by the typical waxing and waning of tics, initial improvements due to the other interventions (e.g., psychoeducation and relaxation exercises), as well as non-specific treatment effects at the beginning of a treatment which may arise from positive expectations and feeling understood by a specialized therapist (e.g., Frank and Frank, 1991; Grawe and Grawe-Gerber, 1999; Viefhaus et al., 2019). A clear superiority of intervention phase (B) (training of tic reaction detection, training of perception of the premonitory urge, and the competing response training) was not found.

In summary, especially three of the five patients (patients 1, 3, and 5) improved during the course of the treatment. Moreover, the clinical impression is that patient 4 improved as well, even though parents and patient did not report improvements. This patient was technically able to control almost all tics for more than half an hour, which is considered as a great improvement, but his motivation to control tics in situations other than during online coaching sessions was lacking. More tic-training sessions would probably not have led to larger improvements, while increasing motivation to use learned strategies would have been an additional therapy goal. However, given that the patient found ERP to be a good strategy for himself and used it to control the tics, one could argue that he did not complete the treatment according to protocol.

Patient 2 showed no improvement; a symptom increase was even found. Two very strong tics emerged during the intervention phase (B), which reduced the treatment motivation. Furthermore, the high degree of confrontation due to the high session frequency and the large number of questionnaires to be completed was probably stressful for this patient. Moreover, the clinical impression was that a regular face-to-face contact was very important for this patient in order to improve the therapeutic relationship. Therefore, the number of face-to-face contacts was increased at the end of the therapy.

An effect size of −2.41 across all patients was found for the self-rated Tic Score (SCL-TIC-S), indicating a large increase of tics. Especially in patient 1, the sharp increase in tic symptoms at t5 is noteworthy, and did not emerge in the other ratings. The clinical impression is that the self-awareness training increased the awareness of the tics. This was indeed intended, but was also accompanied by a perfectionism in tic detection, which may have led to the higher rating at the end. Furthermore, the internal consistency of the SCL-TIC-S is not satisfactory (Döpfner and Görtz-Dorten, 2017), and it is designed for children aged 11 and above. Four of the patients in this study were younger than 11 years and might have had difficulties in introspection (especially before self-awareness training).

Interestingly, the CGI-I was rated with “1 = very much improved” in patient 1, while other ratings did not show such a large improvement in this patient, or even reflected increased symptoms (self-rating). One could argue that the clinician was biased, as she was also the treating therapist. At the end of the study, only very few tics were observed by the clinician. Moreover, parents and patient reported the ability to control the tics in almost all situations. The patient showed large improvements during online coaching and self-guided training sessions. For example, tic 1 was rated by the patient with up to 84 tics per 30 min at the beginning, and after training the competing response she was able to control the tic completely for more than 30 min. However, the frequency ratings of most of the outcome measures (e.g., YGTSS and SCL-TIC) assess wider categories; hence, the achievement is only reflected in a small improvement regarding the total tic scores, while the treating therapist rated the achievement of the patient in terms of controllability and improvement in frequency as a large improvement.

Of further note, patients 2 and 4 benefited the least regarding tic symptoms, and at the same time also showed the worst ratings regarding telepresence and treatment satisfaction. Hence, further studies should also focus on the questions of how these variables are related and whether, or in which way, they can be modified to improve treatment outcome.

In addition, only two patients had a relative improvement of more than 25% (categorized as a clinical success) at therapy end, while at the follow-up assessment four patients had an improvement of at least 25%. Earlier HRT trials using face-to-face interventions often reported a mean reduction of about 30% (e.g., Piacentini et al., 2010; Viefhaus et al., 2018), while the present online coaching study found a mean reduction of 13% at therapy end and of 43% at the follow-up assessment.

Accordingly, the improvement at the 3-month follow-up assessment was larger than the improvement at the end of the treatment (t5). The clinical impression was that three patients were highly motivated to learn to control as many tics as possible before the end of the study, and made further achievements during the final week of online coaching. As the final assessment point was on the day after, or at most a few days after, the final online coaching session, and the measures (e.g., YGTSS) asked about the tic occurrence during the last week, not all changes were assessed at t5 due to the overlapping period. Heijerman-Holtgrefe et al. (2020) addressed this issue by conducting the final assessment point 1 week after the end of treatment. As the patients in the present study had only a small number of sessions during the follow-up phase and the main focus of these sessions was not on HRT strategies, the effects cannot merely be attributed to further therapy sessions. Rather, it is more likely that patients trained on their own and implemented the learned strategies in their daily routine.

Despite some limitations of the present study [no clear superiority of intervention phase (B), not all patients benefited, no improvement for all outcome measures, stronger effects at follow-up assessment than at treatment end], our findings provide first hints for the effectiveness of blended therapy (HRT) with added online coaching in HRT regarding tic symptoms, impairment, and controllability in some patients.

In addition to the improvements regarding tic symptoms and impairment, these first results demonstrate a good feasibility of online coaching. Apart from some internet connection problems and occasional image and sound interference, the transmission quality was sufficient for most patients. Video quality is especially important in the treatment of tics, as the therapist has to observe, detect and differentiate between the tics correctly. In self-awareness training, the direct corrective feedback of the therapist is of particularly great importance, as any delay in video exchange can affect the therapy process and, in the long-term, the therapy success (cf. Himle et al., 2010; Himle et al., 2012).

Most patients were highly motivated for the online coaching, and it was possible to carry out the sessions regularly, reliably and on time with all patients. All tics were sufficiently visible or audible. Only one patient had a vocal tic which was very quiet, which thus required greater effort and awareness on the part of the therapist. Furthermore, with respect to vocal tics, the source of noise was sometimes difficult to identify (e.g., was it the child who coughed or the mother?). It is likely more difficult to train tics which are more concealed (e.g., tics with the feet); however, even for these tics, online coaching might be a good addition if the view frame of the camera is adjusted accordingly. Nevertheless, as emerging tics are more difficult to identify during online coaching sessions, blended therapy may be beneficial. The high frequency of sessions can be advantageous, but also requires high motivation due to its time-consuming nature. Thus, for some patients, the high frequency of HRT might be too confrontational. Furthermore, it is easier to avoid difficult topics in online sessions than in face-to-face-sessions, and misunderstandings in communication are more likely, as non-verbal communication and reading body language are hindered (Storch et al., 2011; Lichstein et al., 2013; Ricketts et al., 2016). However, some patients seem to feel safer at home, while in other cases, regular face-to-face contact (blended therapy) seems to be relevant for the therapeutic relationship (Storch et al., 2011).

Moreover, the combination of face-to-face sessions and videoconferencing has the advantage of reducing travel requirements while still enabling direct contact with the therapist. Although there are no time and cost savings for the therapist, patients have the option to be treated by a specialized therapist. In the present study, HRT *via* videoconference offered a high flexibility regarding session frequency (multiple times per week) and length of the sessions (adaptation to specific needs of the patients and therapy phase). However, not all therapists might be able to offer such flexibility due to their own time schedule.

Another advantage was the high flexibility and possibility to choose different activities during online coaching. In many children, tics increased after school and one child had a tic increase in the evening; hence, sessions were planned during these periods. Due to the long travel distances for the families, sessions at the clinic would not have been possible during these specific periods.

Some children showed specific tics only rarely in face-to-face sessions or while talking with the therapist during online coaching. Specific situations and actions to induce or increase tics for practicing HRT were, for example, playing Nintendo PlayStation, playing online games on their own or with the therapist, watching TV from their couch at home, watching YouTube videos using their tablets, playing with their toys in their room or reading their favorite book to the therapist. For example, one child had a strong tic increase when playing video games with his brother, and therefore during online coaching, he and his brother played these video games together. Moreover, one girl had one specific tic very frequently after she had been watching videos for a longer time period (e.g., 15 min) on her couch at home. Therefore, she started to watch the videos before the online coaching began, and when the therapist joined and the online coaching started, the tics were more frequent and HRT could be practiced.

Another advantage was that the video was sometimes helpful feedback when practicing the competing response. For example, one child was able to use the competing response, but it was noticeable when she was using it because she looked very tense. After this was pointed out to her, she used her video image during online coaching as a kind of mirror, thus providing direct feedback to adjust the competing response, and was able to carry it out much more naturally.

To sum up, online coaching offered a high flexibility in spontaneously inducing tics in naturalistic settings. The clinical impression is that the online coaching was helpful for the implementation into daily routine and in the long-term for generalizing therapy effects.

Nevertheless, some specific characteristics of videoconferencing need to be kept in mind. For example, more communication is needed about what the therapist is doing, because depending on the view frame, patients are unable to fully observe the therapist (e.g., if he/she is writing something down). Furthermore, eye contact is changed, as the camera is usually mounted higher than the location of the therapist’s gaze when he/she is watching the patient. Good technical equipment is needed (e.g., camera), and the families sometimes require technical support (cf. Himle et al., 2012). In addition, therapists need to exercise caution regarding data protection and need to plan how to deal with potential crises of the patient (cf. Himle et al., 2012).

In the present study, patients and parents stated that the online coaching was very helpful (especially patients 1, 3, and 5), and although it was sometimes stressful due to the high frequency of sessions, therapy goals were present, and patients trained and learned a lot in a short period. Furthermore, three families reported high generalization benefits due to the natural setting. For example, one family mentioned that online coaching motivated the patient to practice even more at home. Patients and parents reported feeling comfortable with receiving HRT *via* videoconferencing and four families stated that they liked the combination of face-to-face sessions and online coaching; only one patient would have preferred face-to-face sessions at the end of the treatment.

Some principal limitations of this study have to be taken into account. One main limitation is the small sample size and the two-phase AB design. To enhance scientific credibility, the intervention was replicated across five patients (Levin and Evmenova, 2014). However, a multiple-baseline design, e.g., including varying durations of the intervention (A) phase, would have enabled us to rule out more threats to validity. Another important limitation is the lack of blinded clinical ratings. Overall, further investigations are necessary, including randomized controlled trials and studies with larger sample sizes.

Despite these limitations, some advantages of this study should be emphasized. Multiple outcomes were assessed by clinicians, parents and patients, which is of great importance as the results differ between the raters. Moreover, patient ratings and controllability ratings are so far understudied (Woitecki and Döpfner, 2011; Viefhaus et al., 2018). Furthermore, tic symptoms were systematically observed during online coaching and assessed daily. Single-case analyses allow detailed descriptive analyses of the patients’ course. In addition, a 3-month follow-up was obtained in order to check the stability of the effects. To the best of our knowledge, this is the first study to examine the effects of the combination of face-to-face therapy and video-delivered therapy (blended therapy) in children with chronic tic disorders.

Therefore, despite some principal limitations, the present study provides first hints that blended therapy is feasible and improves symptoms in some children with tics.

## Data Availability Statement

The raw data supporting the conclusions of this article will be made available by the authors, without undue reservation.

## Ethics Statement

The studies involving human participants were reviewed and approved by the Ethics Committee of the University Hospital, Cologne. Written informed consent to participate in this study was provided by the participants’ legal guardian/next of kin.

## Author Contributions

PV was involved in the development of the design, served as the primary therapist for the patients, analyzed and interpreted the data, and drafted the manuscript. JA had been involved in the development of the design and data analysis. HG and KW were involved in the development of the design, recruited study participants, and supervised the treating therapists. MD was involved in the development of the design, analyzed and interpreted the data, and drafted the manuscript. All authors were involved in the preparation of the manuscript, read, and approved the final manuscript.

## Conflict of Interest

KW and MD are authors of the treatment manual evaluated and of other books about tic disorders for which they receive royalties from Hogrefe. The remaining authors declare that the research was conducted in the absence of any commercial or financial relationships that could be construed as a potential conflict of interest.

## Publisher’s Note

All claims expressed in this article are solely those of the authors and do not necessarily represent those of their affiliated organizations, or those of the publisher, the editors and the reviewers. Any product that may be evaluated in this article, or claim that may be made by its manufacturer, is not guaranteed or endorsed by the publisher.
